# Fish oil supplementation reduces osteoarthritis-specific pain in older adults with overweight/obesity

**DOI:** 10.1093/rap/rkaa036

**Published:** 2020-07-23

**Authors:** Julia C Kuszewski, Rachel H X Wong, Peter R C Howe

**Affiliations:** r1 Clinical Nutrition Research Centre, School of Biomedical Sciences and Pharmacy, University of Newcastle, Callaghan, New South Wales; r2 Institute for Resilient Regions, University of Southern Queensland, Springfield Central, Queensland; r3 School of Health Sciences, University of South Australia, Adelaide, South Australia, Australia

**Keywords:** osteoarthritis, obesity, fish oil, curcumin, microvascular function

## Abstract

**Objectives:**

OA is a leading cause of chronic pain and disability. Next to inflammation, vascular pathology has been hypothesized to play a role in its aetiology and progression. Owing to side effects and the low efficacy of pharmacological treatments, dietary supplements are popular as alternative treatments, but evidence of efficacy is limited. We tested whether fish oil and curcumin supplementation can reduce chronic pain and OA burden in older adults.

**Methods:**

A 16-week randomized, double-blind, placebo-controlled, 2 × 2 factorial design supplementation trial with fish oil (2000 mg/day docosahexaenoic acid + 400 mg/day eicosapentaenoic acid), curcumin (160 mg/day) or a combination of both was undertaken in sedentary overweight/obese older adults. Secondary outcomes included treatment-induced changes in self-reported chronic pain and OA burden and whether changes were related to changes in small artery elasticity (surrogate marker for microvascular function), CRP (inflammatory marker) and well-being.

**Results:**

The majority of participants (131 of 152) reported chronic pain, which was predominantly OA specific. Fish oil significantly reduced OA-specific pain (*P* = 0.002, Cohen’s *d* = 0.56) and burden (*P* = 0.015, Cohen’s *d* = 0.45) compared with no fish oil treatment; reductions were correlated with improvements in microvascular function and well-being. Curcumin, alone or in combination with fish oil, did not reduce pain measures.

**Conclusion:**

Our findings indicate potential for fish oil to alleviate OA pain and burden in overweight/obese older adults. Further investigations should be undertaken in patients with clinically diagnosed OA to evaluate fish oil alone and as an adjunct to conventional pharmacotherapy and to investigate underlying mechanisms.

**Trial registration:**

Australian and New Zealand Clinical Trials Register, https://www.anzctr.org.au/Trial/Registration/TrialReview.aspx?id=370788, ACTRN12616000732482p.

**Key messages**

- Docosahexaenoic acid-rich fish oil supplementation alleviated chronic OA pain and burden in sedentary overweight/obese older adults.
- The reductions in OA pain and burden were accompanied by improvements in well-being.
- Reductions in OA pain might be mediated, at least in part, by improvements in microcirculatory function.

## Introduction

OA is a chronic and progressive condition affecting the whole joint and is characterized by remodelling of the subchondral bone, cartilage damage and inflammation of the synovial tissue and tendon [1]. With 18% of women and 9.6% of men >60 years of age affected worldwide, OA is a leading cause of chronic pain and disability in the elderly and the predominant condition for knee and hip replacement, thereby contributing to greater costs and burden on the health-care system [2–4]. OA often co-occurs with obesity, and they can negatively affect one another, resulting in a vicious cycle owing to physical inactivity/disability [5, 6]. Excess weight increases joint loading which, together with the obesity-associated chronic low-grade inflammation and subsequent release of pro-inflammatory cytokines and adipokines, affects the structural integrity of joint bone and cartilage [6]. The link between obesity and OA might also be attributable, in part, to microvascular pathology, which has been hypothesized to play a role in the initiation and progression of joint disease [7, 8]. One hallmark of OA is remodelling of the subchondral bone, which is a highly vascularized tissue. Vascular disease in the subchondral bone is believed to accelerate the OA process owing to altered cartilage nutrition and direct ischaemic effects on the bone [7].

Conventional therapy for OA is medication with NSAIDs; however, their therapeutic efficacy is minimal and is limited by side effects associated with long-term use. Therefore, dietary supplements are gaining increasing popularity as an alternative treatment modality [9, 10]. Two of these are fish oil, containing the long-chain omega-3 polyunsaturated fatty acids (LCn-3PUFAs) docosahexaenoic acid (DHA) and eicosapentaenoic acid (EPA), and curcumin, a polyphenolic compound and the main active component of the curry spice turmeric (*Curcuma longa*). Fish oil and curcumin are both potent anti-inflammatory bioactive nutrients [11]; therefore, benefits for patients with OA are assumed. However, clinical evidence is still inadequate. A meta-analysis by Senftleber *et al*. [12] concluded that LCn-3PUFAs have favourable effects in RA, but evidence for OA pain alleviation is of low quality, because only five randomized clinical trials were identified, and they had a high risk of bias. For curcumin, clinical trials are also limited in number and focus primarily on knee OA. A meta-analysis by Onakpoya *et al*. [13] concluded that curcumin supplementation might have some beneficial effects on knee pain, but pain relief is less effective than with ibuprofen.

We recently reported the effects of a 16-week supplementation trial of fish oil and/or curcumin on systemic and cerebral vascular function (with the primary outcome of cerebrovascular responsiveness to hypercapnia) and inflammation (CRP) in overweight/obese sedentary older adults [14]. In the same study, we tested the effects of fish oil and curcumin, independently and in combination, on self-reported chronic pain and OA burden, and whether any reductions in chronic pain would be related to enhanced microvascular function and reduced inflammation and accompanied by improvements in well-being (including general health perception, mood and depressive symptoms). The aim of this secondary analysis was to provide more clinical evidence on whether fish oil and/or curcumin supplementation could be used as an alternative treatment modality for OA pain and burden in a population group at increased risk of developing OA.

## Methods

### Study design

A 16-week randomized, double-blind, 2 × 2 factorial, placebo-controlled intervention was undertaken at the University of Newcastle’s Clinical Nutrition Research Centre. Community-dwelling adults residing in the Hunter region of New South Wales aged between 50 and 80 years with overweight or obesity (BMI 25–40 kg/m^2^) who had a sedentary lifestyle (<150 min of planned physical activity per week) were recruited via approved media advertising (radio interviews, newspaper articles and website advertisements) and by contacting local social groups for older adults. Participants were excluded if their fish/seafood intake exceeded more than two servings per week or >300 mg/day of LCn-3PUFAs from fish oil supplements, if they had suspected dementia, had been diagnosed with major depression, had a history of cardiovascular, kidney or liver disease or a neurological condition or were on insulin or warfarin therapy. This study was approved by the University of Newcastle’s Human Research Ethics Committee (H-2016-0170), registered with the Australian and New Zealand Clinical Trials Register (ACTRN12616000732482p) and conducted in accordance with the International Conference on Harmonization Guidelines for Good Clinical Practice. Written consent was obtained before commencement.

An independent investigator allocated participants to one of the following four treatment groups according to the Altman’s allocation by minimization method [15] to ensure balanced groups based on age, BMI and sex: fish oil (FO; total dose of 400 mg EPA and 2000 mg DHA); curcumin (CUR; total dose of 800mg Longvida® containing 160mg curcumin); fish oil and curcumin (FO+CUR; 400 mg EPA, 2000 mg DHA and 160 mg curcumin); or matching placebos (PL), either FO placebo [mix of corn oil and olive with 20 mg of fish oil (odour match)] or CUR placebo (maltodextrin with yellow food colouring).

Capsules were supplied by Blackmores Institute (Sydney, NSW, Australia) and were identical in appearance to their respective placebos, identifiable only by code. The active fish oil capsules (Blackmores Omega Brain) contained 100 mg EPA and 500 mg DHA each, and the active curcumin capsules (Blackmores Brain Active) contained 400 mg Longvida extract (80 mg curcumin). During the 16-week intervention, participants were instructed to consume six capsules daily (two fish oil and one curcumin or matching placebo, both morning and evening with meals) and to maintain their habitual diet and exercise regimen. To facilitate compliance, participants were asked to record each supplement intake in an assigned diary together with any changes in their medication intake and were followed up with a phone call at mid-intervention to enquire about their well-being. At the end of the trial, all remaining capsules were counted to assess overall compliance. Blinding was maintained until all data analysis had been completed.

### Study procedures and outcome measures

A detailed description of the study procedures has been published [14]. Briefly, participants attended the research facility a total of four times (two in the beginning and two at the end of the intervention), having refrained from medication, food and beverages other than water for ≥2 h before the visit. During the first visit, anthropometric measures were taken, and participants were screened for suspected dementia (<82/100 on the Addenbrooke’s cognitive examination III). Then, participants were seated down for ≥10 min before measurement of arterial compliance (Cardiovascular Profiler CR2000; Hypertension Diagnostics Inc.). Arterial pulse waveform analysis was used to assess the artery elasticity index of the small arteries, an indicator for endothelial dysfunction. Three consecutive measurements were taken and averaged for analysis. During the second visit, a venous blood sample was collected by a commercial pathology centre after an overnight fast to measure the inflammatory biomarker high-sensitivity CRP. Then, participants were asked to complete questionnaires regarding their chronic pain and OA burden, in addition to their general well-being. All assessments were repeated in the same order at the end of the 16-week trial.

#### Chronic pain measures

The participants’ chronic pain symptoms (>1 month) were captured with the short-form McGill Pain Questionnaire, which consists of three parts [16]. Initially, participants were asked to assign an intensity to each of the 15 descriptors of sensory pain using a Likert scale ranging from none to severe (pain score). Each intensity scale was assigned a numerical value, with 45 being the maximum total score, indicating most pain. Furthermore, chronic pain intensity was assessed using a 10 cm visual analog scale (VAS), and a five-point scale ranging from no pain to excruciating was used to measure present pain intensity (PPI). Each subscale (pain score, VAS and PPI) score was expressed as a percentage, and an average of all subscales was taken to give an overall pain score. Moreover, pain location was captured using a body diagram.

The OA Questionnaire (OA-Quest) was used to assess the impact of chronic pain attributable to OA on the participant’s daily life during the last 4 weeks before their scheduled visit [17]. The questionnaire consisted of 42 statements and included seven dimensions of OA burden (i.e. physical distress, sleep disturbance, psychological distress, loss of productivity, physical limitations, physical deconditioning and financial hardship). Participants had to indicate how true each of the statements was for them on a Likert scale ranging from not at all to extremely, with each scale being assigned a numerical value. A sum of all values was taken and expressed as a percentage of the maximum score (168 points), with a higher score indicating more OA burden.

#### Well-being questionnaires

The participants’ perception of physical and mental well-being during the last 4 weeks before their scheduled visit was assessed with the 36-Item Short Form Survey (SF-36), which includes nine subscales [18]. Each subscale has a maximum score of 100, indicating no disability, and the overall perception of quality of life (QoL) was calculated by taking an average of all subscale scores.

The participants’ mood states over the last 7 days before their scheduled visit were assessed with the Profile of Mood states questionnaire. The 65 descriptive words were divided into six mood subscales, each of which was expressed as a percentage of the maximum score. The total mood disturbance (TMD) was calculated by summing the percentages of all subscales (except vigour), dividing by the maximum score and subtracting the percentage obtained for vigour. A more negative TMD percentage indicates better overall mood.

Depressive symptoms were assessed with the Centre for Epidemiologic Studies Depression Scale (CES-D) questionnaire. It consists of 20 statements, and participants had to rate the frequency of their experience for each statement during the last 7 days before their visit (from rarely to most or all the time). Each frequency was assigned a numerical score, with a maximum score of 60, which was expressed as a percentage. A percentage of ≥25 indicates a risk of depression.

### Statistical analysis

This is a retrospective exploratory analysis of secondary outcomes in our previously published clinical trial [14]. To detect a 0.7 effect size (Cohen’s *d*) difference between treatment groups in the primary outcome (cerebrovascular responsiveness to hypercapnia) at α = 0.05, an estimated 136 participants were needed. 

Using a per-protocol analysis and setting compliance to 80%, differences in changes in chronic pain measures between each treatment group and placebo were determined by ANOVA (IBM SPSS v.24; New York, NY, USA), with baseline pain (model 1) or baseline pain and BMI (model 2) as covariates. Moreover, using the 2 × 2 factorial design, effects of fish oil and curcumin treatment were assessed independently, as follows: fish oil (FO and FO+CUR groups) *vs* no fish oil (CUR and PL groups); and curcumin (CUR and FO+CUR groups) *vs* no curcumin (FO and PL groups).

Pearson’s correlation analysis was used to determine whether changes in chronic pain symptoms were related to changes in the small artery elasticity index (used herein as a surrogate marker of the microvasculature), CRP, QoL, overall mood and depressive symptoms. All results are presented as the mean ± s.e.m.

## Results

### Participants and baseline pain measures

Between June 2017 and August 2018, 152 participants were enrolled, of whom 134 completed the intervention, and after setting the compliance to 80%, 126 participants remained (for CONSORT diagram, see Kuszewski *et al*. [14]). Side effects were reported in four participants (digestive problems: PL *n* = 1, CUR *n* = 1 and FO+CUR *n* = 1; reflux: PL *n* = 1), but no serious adverse events were reported. Two participants were excluded from the pain analysis (PL *n* = 1 and FO+CUR *n* = 1), owing to unrelated pain that was non-existent at baseline (shingles), leaving 124 participants for analysis (PL *n* = 31, FO *n* = 32, CUR *n* = 31 and FO+CUR *n* = 30).

The baseline characteristics of participants are described in Table 1. Chronic pain measures were not significantly different between groups except for OA burden, which was significantly lower in the FO group compared with the CUR group (*P* = 0.021). One hundred and thirty-one participants (86%) reported chronic pain (McGill Pain Questionnaire) at baseline. For the majority (70%), however, pain symptoms were mild, as determined by VAS, averaging 3.5 ± 0.2/10 cm [19]. The pain was mostly OA specific, because it was predominantly reported in the knees (41%), lower back (39%) and shoulders (31%), with aching being the most common pain descriptor (83%). Furthermore, overall pain and pain subscales were correlated strongly with OA burden, with most participants reporting physical deconditioning/limitation (reduction of physical exercise, difficulties with stairs), sleep disturbance and physical distress (weakened joints) (OA-Quest).

**Table 1:** Baseline characteristics of participants in each group and their correlations with overall pain and OA burden at baseline

	Group				Correlation			
Characteristics	Placebo	Fish oil	Curcumin	Fish oil + curcumin	Overall pain		OA burden	
	(*n* = 36)	(*n* = 39)	(*n* = 38)	(*n* = 39)	*r*	*P*-value	*r*	*P*-value
Age, years	65.4 ± 1.3	65.4 ± 1.2	65.4 ± 1.2	66.2 ± 1.3	−0.161	0.048	−0.124	0.128
BMI, kg/m^2^	31.0 ± 0.7	31.0 ± 0.7	30.5 ± 0.7	30.9 ± 0.6	0.356	<0.001	0.288	<0.001
Waist, cm	105.4 ± 2.0	105.5 ± 1.6	103.8 ± 2.0	104.4 ± 1.8	0.291	<0.001	0.254	0.002
Physical activity, min/week	33.8 ± 9.7	55.1 ± 12.8	44.4 ± 13.0	54.6 ± 14.3	−0.066	0.443	−0.155	0.068
CRP, mg/l	2.2 ± 0.4	2.3 ± 0.3	2.6 ± 0.5	2.3 ± 0.2	0.149	0.067	0.144	0.162
Quality of life (score/100)	70.4 ± 2.4	72.5 ± 1.9	67.1 ± 2.5	69.4 ± 2.4	−0.610	<0.001	−0.663	<0.001
Depressive symptoms, %	13.4 ± 1.9	14.0 ± 1.7	17.0 ± 2.1	15.4 ± 2.4	0.379	<0.001	0.510	<0.001
Total mood disturbance, %^a^	−40.1 ± 3.6	−33.7 ± 4.6	−28.7 ± 4.2	−33.0 ± 5.0	0.259	<0.001	0.362	<0.001
Overall pain, %	25.5 ± 3.3	21.0 ± 2.0	25.1 ± 3.1	22.9 ± 2.5	–	–	0.714	<0.001
Pain score	13.7 ± 2.4	9.6 ± 1.4	14.6 ± 2.9	12.4 ± 2.4	–	–	0.698	<0.001
VAS	34.4 ± 5.0	26.2 ± 3.1	33.8 ± 4.0	29.7 ± 3.7	–	–	0.637	<0.001
PPI	28.3 ± 3.2	27.2 ± 2.5	26.8 ± 3.6	26.7 ± 2.9	–	–	0.580	<0.001
OA burden (%)	11.2 ± 2.6	5.8 ± 1.2	12.6 ± 2.4	9.8 ± 1.8	–	–	–	–

Data are presented as the mean ± s.e.m.

a Greater negative value (maximum: −100) indicates better overall mood.

PPI: present pain intensity; VAS: visual analog scale.

Participants with greater chronic pain symptoms and OA burden had a higher BMI, lower QoL and more depressive symptoms and mood disturbance. However, pain symptoms and OA burden were independent of inflammation (CRP).

### Treatment-induced changes in measures of pain

Fish oil supplementation for 16 weeks significantly reduced the pain score and PPI (both *P* = 0.012, Cohen’s *d* = 0.65) compared with placebo, resulting in a 42% reduction of overall chronic pain (*P* = 0.013, Cohen’s *d* = 0.64) (Table 2). Furthermore, it tended to reduce the OA burden by 40% (*P* = 0.052, Cohen’s *d* = 0.49), which reached significance after adjusting for baseline BMI (model 2; *P* = 0.050, Cohen’s *d* = 0.50, compared with placebo). In contrast, curcumin supplementation did not affect any chronic pain measures. The combination of fish oil and curcumin tended to reduce chronic pain measures, but only the reduction of PPI was significant (*P* = 0.041, Cohen’s *d* = 0.12).

**Table 2:** Treatment-induced change in individual outcome measures of pain, expressed as percentages, per group

	Placebo (*n* = 31)	Fish oil (*n* = 32)	Curcumin (*n* = 31)	Fish oil + curcumin (*n* = 30)
Overall pain				
Model 1	−0.4 ± 2.4	−8.9 ± 2.4^*^	2.9 ± 2.4	−3.5 ± 2.4
Model 2	−0.4 ± 2.4	−8.9 ± 2.4^*^	3.0 ± 2.4	−3.5 ± 2.4
Pain score				
Model 1	0.2 ± 1.5	−5.4 ± 1.6^*^	3.4 ± 1.6	−4.3 ± 1.6^*^
Model 2	0.2 ± 1.5	−5.4 ± 1.6^*^	3.3 ± 1.6	−4.4 ± 1.6^*^
VAS				
Model 1	1.9 ± 3.7	−7.4 ± 3.7	2.9 ± 3.7	−1.6 ± 3.8
Model 2	1.9 ± 3.7	−7.5 ± 3.8	3.0 ± 3.7	−1.5 ± 3.8
PPI				
Model 1	−2.8 ± 3.0	−13.7 ± 3.0^*^	2.4 ± 3.0	−4.9 ± 3.1
Model 2	−2.9 ± 3.0	−13.8 ± 3.0^*^	2.6 ± 3.0	−4.8 ± 3.0
OA burden				
Model 1	1.0 ± 1.2	−2.3 ± 1.2	0.6 ± 1.2	−2.0 ± 1.2
Model 2	1.0 ± 1.2	−2.4 ± 1.2^*^	0.6 ± 1.2	−2.0 ± 1.2

Model 1: adjusted for baseline pain; model 2: adjusted for baseline pain and BMI. Data are presented as the mean ± s.e.m.

* Statistically significant from placebo (*P* < 0.05).

PPI: present pain intensity; VAS: visual analog scale.

**Fig. 1:**
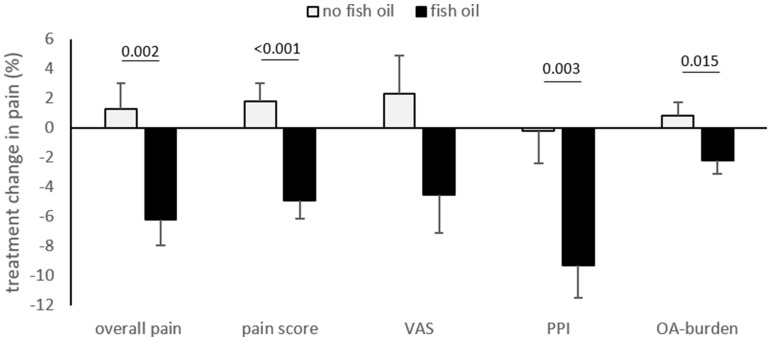
Treatment-induced changes in individual pain measures after fish oil *vs* no fish oil treatment Baseline pain and BMI were used as covariates; *n* = 62 per group. PPI: present pain intensity; VAS: visual analog scale.

Using the 2 × 2 factorial analysis, fish oil treatment significantly reduced the pain score (*P* < 0.001, Cohen’s *d* = 0.85), PPI (*P* = 0.003, Cohen’s *d* = 0.54) and overall pain (*P* = 0.002, Cohen’s *d* = 0.56), in addition to the OA burden (*P* = 0.015, Cohen’s *d* = 0.45) compared with no fish oil treatment (Fig. 1). The reduction in overall pain and OA burden in participants supplemented with fish oil was greater in those who reported higher baseline pain/OA burden at baseline (*n* = 62; pain: *r* = −0.528, *P* < 0.001; OA burden: *r* = −0.548, *P* < 0.001).

**Fig. 2:**
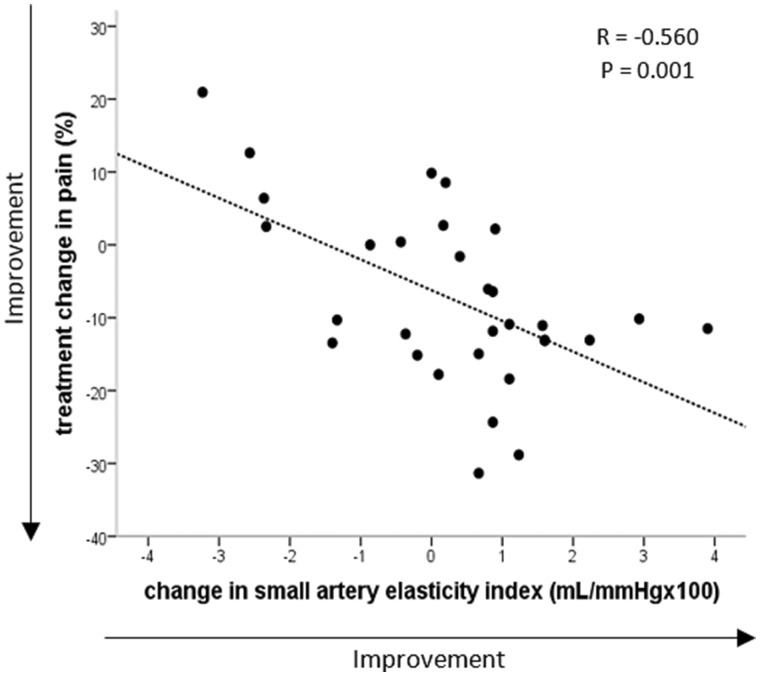
Correlation between change in overall pain and small artery elasticity in participants randomized to fish oil *n* = 32.

The reductions in overall pain after fish oil treatment were associated with increases in small artery elasticity index (Fig. 2; FO group only: *n* = 32, *r* = −0.560, *P* = 0.001; FO 2 × 2 factorial design: *n* = 62, *r* = −0.296, *P* = 0.022), but not with changes in CRP (*n* = 62, *r* = −0.167, *P* = 0.211).

Treatment-induced changes in pain measures reported above included all participants who finished the intervention with a compliance of >80%. We also performed a separate analysis with only those participants who reported chronic pain at baseline (*n* = 131), but the outcomes were unaffected.

### Correlations between changes in chronic pain and changes in well-being

**Fig. 3:**
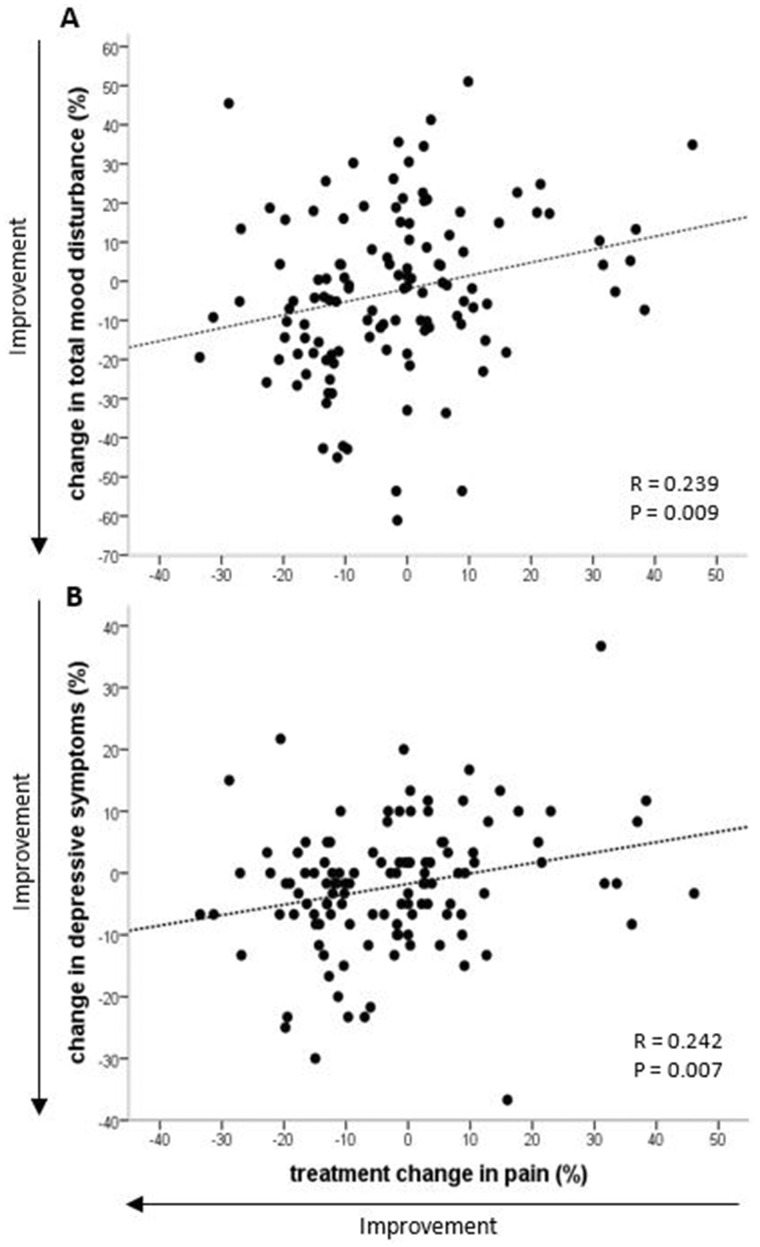
Correlation between change in total mood disturbance (**A**) or depressive symptoms (**B**) and overall pain *n* = 121.

The reduction in overall pain was correlated with reductions in TMD (*r* = 0.239, *P* = 0.009) and depressive symptoms (*r* = 0.242, *P* = 0.007) (Fig. 3). Furthermore, the reduction in overall pain tended to be correlated with better perception of total health (QoL; *r* = −0.177, *P* = 0.055) and was correlated significantly with perception of physical health (*r* = −0.188, *P* = 0.040). Similar correlations were observed between changes in OA burden and TMD (*r* = 0.270, *P* = 0.003), depressive symptoms (*r* = 0.286, *P* = 0.001) and QoL (*r* = −0.336, *P* < 0.001).

## Discussion

In this exploratory analysis, 16 weeks of supplementation with DHA-rich fish oil but not curcumin reduced mild chronic pain symptoms, which were predominantly OA specific, in addition to OA burden in overweight/obese sedentary older adults. These reductions were accompanied by enhanced well-being (i.e. reductions in mood disturbance and depressive symptoms and an improvement in physical health perception, independent of changes in BMI).

### Potential mechanisms underlying the pain-reducing effects of fish oil

Current treatments for OA target inflammation, because OA is associated with increases in several pro-inflammatory cytokines, such as IL-1, that are involved in cartilage degradation, which is one of the causes of OA pain symptoms [20, 21]. Owing to the well-known anti-inflammatory effects of fish oil [22], it was expected to be beneficial in OA treatment [10]. However, since 1989 only five studies have examined the effect of marine oils (containing LCn-3PUFAs) on OA-specific pain, of which three showed slight reductions in pain, but the quality of those studies was very low owing to high risk of bias [12]. The studies examined CRP levels, but found no significant treatment-induced changes. There is evidence from *in vitro* studies showing that fish oil can reduce IL-1 in bovine chondrocytes and decrease inflammation-induced destruction of tissue in human OA cartilage harvested during surgical procedures [10]. In our study, fish oil supplementation slightly reduced the inflammatory marker CRP [14], but the reduction was not significant and was not correlated with a reduction in pain symptoms. However, one limitation of this study is that we did not measure other inflammatory markers involved in OA, such as IL-1 or TNF-α, which might have been more sensitive. Furthermore, participants reported mild chronic pain and OA burden, and CRP levels were only slightly elevated (<3 mg/l), suggesting that their OA was still in the early stages, with minimal cartilage degradation. Further well-designed studies are needed in participants with higher CRP levels and more advanced radiographic and symptomatic OA to test the hypothesis that fish oil reduces OA pain by counteracting inflammation.

Another cause of pain in OA is the reduction in blood flow attributable to vascular disease and consequent damage to the microvasculature between the articular cartilage and subchondral bone [7, 21]. Impaired blood perfusion of tissues can cause ischaemic pain, mediated by the loss of oxygen and nutrient supply to nerves; thus, restoring blood flow is a promising treatment target for pain relief in OA [21]. Our finding of a correlation between reductions of chronic OA-specific pain after fish oil supplementation and increases in the small artery elasticity index suggests that ability of fish oil to alleviate pain might be attributable, at least in part, to improved microvascular function. The endothelium is the key regulator of blood flow, and endothelial dysfunction is characterized by impaired nitric oxide production or availability, leading to impaired endothelium-dependent vasodilatation [23]. Fish oil can improve endothelial function by increasing production of the vasodilators nitric oxide and prostacyclin, decreasing production of the vasoconstrictor ET-1 and reducing oxidative stress [24]. Clinical trials have shown that fish oil supplementation can enhance flow-mediated dilatation, a marker of endothelial function [25]. The improved endothelium-dependent vasodilatation after fish oil supplementation might improve perfusion to previously hypoxic tissues, restoring the oxygen and nutrient supply in affected joints, thereby reducing ischaemic pain [21].

### Effects of curcumin supplementation

There is evidence that curcumin inhibits inflammation in bovine and human chondrocytes, and human clinical trials have shown slight reductions in knee OA pain, although the efficacy was lower compared with ibuprofen [13]. In our study, curcumin supplementation did not affect OA-specific pain or the OA burden in overweight/obese older adults. One possible reason might be that the OA pain and burden in our study population was too mild. The previous studies were performed in patients with established OA associated with more severe pain [13]. When we explored the effects of curcumin in a subgroup of participants with moderate-to-severe pain (*n* = 35, VAS: 4.6–9.4 cm), curcumin elicited a slight, non-significant reduction in pain as opposed to a non-significant increase in pain seen in participants with mild chronic pain (*n* = 88, VAS < 4.5 cm). This suggests that curcumin might have potential benefits in adults with moderate-to-severe OA pain but could be disadvantageous in adults with mild OA pain. Furthermore, it should be noted that previous studies were limited to knee OA, whereas our study looked at general OA pain, although knee pain was most common. Further investigations are required to identify OA patients who might benefit from curcumin supplementation based on pain severity, location and, possibly, the absence or presence of metabolic disorders.

### Combination of fish oil with other nutrients or medication

The combination of fish oil and curcumin did not result in additional OA pain relief and seemed to dampen the reduction of OA pain and burden observed with fish oil alone. Combining fish oil with curcumin for additional benefits is a very new approach; clinical trials are limited, with inconsistent results [14, 26–28], and effects on OA pain relief have not yet been tested. In migraine patients, fish oil and curcumin were found to have synergistic effects on reduction of inflammatory markers [26, 27] and migraine attack frequency [27]. However, in people at high risk of developing type 2 diabetes, the combination did not result in any complementary effects on glycaemic control and blood lipids [28], and in our study we did not find any synergistic effects on cardiometabolic risk factors and cerebrovascular function [14]. Further studies are warranted to understand the interaction between fish oil and curcumin in humans and to ascertain whether there is a specific dose combination for fish oil and curcumin for synergistic effects and whether it is beneficial only in specific population groups (i.e. migraine patients).

Clinical trials investigating the combination of fish oil with other supplements or NSAIDs for the reduction of OA symptoms are very limited. Fish oil combined with glucosamine, another popular supplement for OA treatment, did not result in greater pain reduction compared with glucosamine alone [29]. The potential of fish oil as an adjunct to NSAID medication for OA pain relief has been tested in two clinical trials, one of which found no significant benefit [30], whereas the other was poorly designed, with no placebo control and no monitoring of fish oil supplementation [31]. Thus, further well-designed trials should be undertaken to evaluate the potential of fish oil as an adjunct to pharmacological treatment to achieve greater pain relief and reduce the dose of medication required and associated side effects.

### Study limitations and future directions

It is important to note that this is an exploratory analysis of secondary outcomes from a large-scale randomized controlled trial. Thus, participants were not recruited based on the presence or intensity of OA pain, and the study was not powered to detect a difference in pain reduction. Moreover, it should be noted that OA was not diagnosed based on radiographic findings. In this study, we used the short-form McGill Pain Questionnaire, which has been shown to be sensitive to demonstrate changes in pain attributable to treatment [32], to measure the quality, intensity and location of general chronic pain (lasting >1 month). The description and location of chronic pain indicated whether the reported pain was OA related, which was then confirmed with the OA-Quest (if the participant had no pain or other non-OA-related pain, the OA-Quest score was 0%). Furthermore, the OA-Quest gave important information on how the OA-related pain impacted the daily life of the participants’.

Another limitation is that the basal self-reported OA pain and burden was mild in the majority of participants. Nevertheless, we still observed a reduction in OA-specific pain and burden after fish oil supplementation, which was accompanied by improvements in well-being. Thus, supplementing the diet with fish oil might offer a potential intervention in individuals who are in an early OA stage that might prevent further OA progression. However, further studies are needed to confirm the promising OA pain-reducing effects of fish oil. These should be conducted in individuals with clinically diagnosed OA with pain symptoms ranging from mild to severe and have longer intervention periods (>6 months) with more frequent assessments of pain, especially in the beginning of the trial, to examine when the pain reduction commences, whether the pain reduction is sustained long term and at which stage of OA progression individuals benefit most from fish oil supplementation.

### Conclusions

Our findings indicate potential for fish oil supplementation to reduce mild OA pain and burden in sedentary overweight/obese older adults with self-reported OA-specific pain, which was associated with improved well-being. The pain-alleviating effects of fish oil might be mediated, at least in part, by improvements in microvascular function. Further studies are warranted to evaluate the benefits of DHA-rich fish oil, alone or as an adjunct to pharmacotherapy, in patients diagnosed with OA who suffer moderate-to-severe pain, to investigate the mechanisms underlying the potential pain-reducing effects of DHA-rich fish oil and to identify OA patients who might benefit from curcumin supplementation.
